# Inhibition of fatty acid-binding protein 4 alleviates psoriasis-like skin inflammation by modulating macrophage polarization

**DOI:** 10.3389/fimmu.2026.1830077

**Published:** 2026-08-03

**Authors:** Bo Mi Kang, Hyun Seung Choi, Bo Ri Kim, Sang Woong Youn, Hyun Jung Kwon, Jin-Ku Lee, Christine Suh-Yun Joh, Hyo Jeong Nam, Soyoung Jeong, Hyun Woo Kim, Jeong Eun Kim, Tae-Gyun Kim, Hyun Je Kim, Chong Won Choi

**Affiliations:** 1 Department of Dermatology, Seoul National University College of Medicine, Seoul, Republic of Korea; 2 Department of Dermatology, Seoul National University Bundang Hospital, Seongnam, Republic of Korea; 3 Institute of Allergy and Clinical Immunology, Seoul National University Medical Research Center, Seoul, Republic of Korea; 4 Department of Biomedical Sciences, Seoul National University Graduate School, Seoul, Republic of Korea; 5 Cancer Research Institute, Seoul National University College of Medicine, Seoul, Republic of Korea; 6 Department of Pathology, Seoul National University Bundang Hospital, Seongnam, Republic of Korea; 7 Department of Biomedical Sciences, Seoul National University College of Medicine, Seoul, Republic of Korea; 8 Department of Anatomy and Cell Biology, Seoul National University College of Medicine, Seoul, Republic of Korea; 9 Department of Dermatology, Hanyang University College of Medicine, Seoul, Republic of Korea; 10 Hanyang Institute of Bioscience and Biotechnology, Hanyang University, Seoul, Republic of Korea; 11 Department of Dermatology, Severance Hospital, Cutaneous Biology Research Institute, Yonsei University College of Medicine, Seoul, Republic of Korea; 12 Institute for Immunology and Immunological Diseases, Yonsei University College of Medicine, Seoul, Republic of Korea; 13 Interdisciplinary Program in Artificial Intelligence (IPAI), Seoul National University, Seoul, Republic of Korea

**Keywords:** fatty acid-binding protein 4, macrophage, macrophage polarization, PPARG, psoriasis

## Abstract

Fatty acid-binding proteins (FABPs) are intracellular lipid chaperones that regulate gene expression by controlling lipid trafficking within subcellular organelles. Among them, FABP4 is primarily expressed in adipocytes and macrophages and has been implicated in inflammatory responses in adipose tissue; however, its role in skin inflammation remains poorly understood. Here, we investigated the immunomodulatory role of FABP4 using human psoriatic skin samples, single-cell RNA sequencing, and an imiquimod-induced mouse model of psoriasis. We further employed pharmacologic inhibition and genetic modulation of FABP4 to assess its effects on macrophage polarization, along with transcription factor regulon analyses to identify downstream regulatory mechanisms. FABP4 was highly expressed in macrophages infiltrating psoriatic skin, and *in vivo* pharmacological inhibition of FABP4 attenuated psoriasiform skin inflammation and shifted macrophage phenotypes, suppressing M1-associated features while promoting CD206-expressing alternatively activated macrophages. For mechanistic insights, we examined the regulon activity of transcription factors associated with macrophage polarization in psoriatic skin and identified an association between PPARG and macrophage polarization; our *in vivo* and *in vitro* experiments further demonstrated that FABP4 inhibition restored PPARG expression and activity in psoriasis mice, which was decreased by imiquimod application. Overall, our study demonstrated that FABP4 plays a critical role in skin inflammation by modulating macrophage polarization through PPARG regulation, suggesting that targeting FABP4 may represent a potential therapeutic strategy for psoriasis and other inflammatory skin diseases.

## Introduction

Psoriasis is a common chronic inflammatory disease. Although psoriasis involves mainly the skin, nails, and joints, previous studies have revealed an association of psoriasis with inflammatory conditions in other organs including inflammatory arthritis, Crohn’s disease, obesity, and metabolic syndrome (1, 2). Among them, the association between obesity and psoriasis has received increased attention: patients with psoriasis have a higher risk of obesity, with the risk increasing with psoriasis severity (3, 4). Additionally, an inverse relationship was also revealed, that is, obese people have a higher risk of psoriasis (3, 5). The association between psoriasis and obesity has been attributed to common inflammatory pathways and metabolic abnormalities (2). Recent clinical and *in-vivo* animal studies found that abnormalities in fatty acid metabolism may play a role in the pathogenesis of psoriasis and its comorbidities (2, 6, 7). For example, in an imiquimod-induced mouse model fed a high-fat diet, increased fatty acids aggravated skin inflammation (6). Regarding the link between fatty acids and inflammation, previous studies have revealed that fatty acids regulate inflammation by modulating immune cells (8). Among them, myeloid cells have been identified as primarily responsible for increasing the inflammatory response and augmenting the activation of keratinocytes in psoriatic mice (9). These results strongly suggest an association between psoriasis, dysregulated fatty acid metabolism, and macrophages.

Fatty acids act as signaling molecules in inflammatory responses and as an energy source for cells (10, 11). When lipids enter cells, fatty acid-binding protein (FABP), an intracellular lipid chaperone, controls the trafficking of lipids in subcellular organelles, thereby regulating gene expression in the nucleus (10, 12). FABP4 is an isoform of FABP highly expressed in adipocytes and macrophages (13, 14). Crucially, previous studies have identified FABP4 not merely as a lipid chaperone but as a central regulator at the interface of lipid metabolism and inflammation, specifically in macrophages (15, 16). In macrophages which abundantly express FABP4, activation of FABP4 plays a major role in macrophage polarization toward the pro-inflammatory M1 subtype by coupling intracellular fatty acid trafficking with the NF-κB and JNK signaling pathways (15, 17, 18). Notably, the role of FABP4 in the regulation of inflammatory reactions has also been demonstrated in animal models and human research (19–21). Considering the increased expression of FABP4 in patients with psoriasis, especially in obese patients (22), the association between macrophage differentiation, FABP4, and fatty acids in psoriasis can be inferred.

Emerging studies have illustrated that patients with psoriasis exhibited elevated FABP4 levels; however, the mechanism by which FABP4 contributes to psoriasis development remains unexplored (23). Given that macrophages are key effector cells that infiltrate psoriatic lesions and orchestrate local inflammation, we hypothesized that FABP4 might link dysregulated lipid metabolism to psoriatic skin inflammation specifically through macrophage modulation. To explore this, we first utilized single-cell RNA sequencing (scRNA-seq) to profile human psoriatic skin, revealing a distinct macrophage polarization imbalance skewed toward a proinflammatory M1 phenotype. To elucidate the upstream regulatory mechanisms governing this polarization, we performed transcription factor regulon analysis and identified peroxisome proliferator-activated receptor gamma (PPARG), which is known to be closely related to FABP4, as a master transcription factor driving the anti-inflammatory M2 macrophage program. Based on these clinical and bioinformatic findings, we investigated the role of FABP4 in an imiquimod-induced psoriasis mouse model and *in vitro* macrophage cultures. Our study demonstrated that both pharmacological inhibition and genetic silencing of FABP4 effectively alleviated skin inflammation by restoring PPARG protein stability and activity, which in turn recalibrated macrophage polarization toward the resolving M2 phenotype.

## Materials and methods

### Animals

For the *in vivo* study, 7-week-old female C57BL/6 mice were purchased (Orient Bio Inc, Gyeonggi, Republic of Korea). Mice were housed in a specific pathogen-free facility with sterile food and water *ad libitum* and were acclimatized for at least 1 week before being used in the experiments. All animal experiments were approved by the Animal Experimental Ethics Committee of our hospital (BA-2208-349-003-04). All procedures were conducted in accordance with the institutional guidelines for the care and use of laboratory animals and complied with the ARRIVE guidelines.

### Induction of imiquimod-induced psoriasis-like skin inflammation in mice

A daily topical dose of 62.5 mg of 5% imiquimod cream (Aldara^®^; 3M Pharmaceuticals, Maplewood, MN, USA) was applied to the shaved backs and ears of 8-week-old female C57BL/6 mice for five consecutive days. BMS-309403, a FABP4 inhibitor, was dissolved in DMSO as a stock solution and diluted in PBS immediately before use. Vehicle and FABP4 inhibitor (0.5 mg/kg and 1 mg/kg; BMS-309403, Medchemexpress LLC, Monmouth Junction, NJ, USA) were administered by intraperitoneal injection into mice on days 2 and 4. During the experiment, the severity of psoriasis-like skin inflammation (PASI score) was assessed using the psoriasis area and severity index (PASI), which is calculated by summing the scores for erythema, scaling, and thickness in the psoriasis area. In addition, ear thickness was measured once daily at the same time point before the daily application of imiquimod using a digital caliper (Mitutoyo, Japan) to assess gross inflammatory changes. On day 6, the mice were euthanized via CO_2_ inhalation, and back and ear skin tissues were collected for histological analysis, quantitative reverse transcription PCR (RT-qPCR), and flow cytometric analysis. According to IACUC ethical standards, if a side effect occurred, such as a decrease in feed intake or a weight loss of >20% of normal body weight, the experiment was terminated. However, no changes in the animals were observed that could be attributed to these experiments.

### Immunofluorescence and histopathologic analyses

For histopathologic analysis, the back and ear skin of mice were harvested and fixed in 4% paraformaldehyde solution. Paraffin-embedded skin tissue sections were deparaffinized and rehydrated using an ethanol series. The sections were stained with hematoxylin and eosin (H&E). A slide scanner (PANNORAMIC 250 Flash III, 3DHISTECH, Budapest, Hungary) was used to acquire the images for histopathological analysis. To evaluate the severity of psoriasis-like skin inflammation, we measured the epidermal thickness using the ImageJ software. Ear thickness measured by digital caliper (Mitutoyo, Japan) reflects overall tissue swelling *in vivo*, whereas epidermal thickness assessed from H&E-stained sections represents histological changes at the tissue level.

For the immunofluorescence analysis, tissue samples were obtained from patients who presented to Seoul National University Bundang Hospital and underwent skin biopsies. This study was approved by the Institutional Review Board of Seoul National University Bundang Hospital (B-2309-851-303). First, the paraffin-embedded tissue sample was cut at 4 µm thickness and deparaffinized. The sections were subjected to heat-induced antigen retrieval in 0.01 M citrate buffer (pH 6.0; Thermo Fisher Scientific, Waltham, MA, USA). After antigen retrieval, the sections were blocked with a blocking solution (Epredia UltraVision Protein Block, #TA-125-PBQ, Thermo Fisher Scientific) for 30 min at 25 °C and incubated with the primary antibodies in a humidified chamber at 4 °C overnight. Primary antibodies against FABP4 were purchased from Thermo Fisher Scientific (1:100 dilution; PA5-30591). For human skin sections, CD68 was used as a macrophage marker, and primary antibodies against CD68 were purchased from Abcam (1:100 dilution; ab201340). For mouse skin sections, F4/80 was used as a macrophage marker, and primary antibodies against F4/80 were purchased from Invitrogen (1:100 dilution; MA1-91124; Waltham, MA, USA). After washing in phosphate-buffered saline (PBS), the sections were incubated with secondary antibody (Alexa Fluor 488 and 594, 1:200 dilution; Invitrogen, Waltham, MA, USA) at 25 °C for 1 h. Last, the sections were stained with 4′-6-diamidino-2-phenylindole dihydrochloride (Thermo Fisher Scientific) at 25 °C for 10 min. The samples were mounted using Faramount Aqueous Mounting Medium (Dako, Santa Clara, CA, USA). Confocal laser scanning microscopy (LSM710; Carl Zeiss, Oberkochen, Germany) was used to acquire the images for immunofluorescence analysis.

### Quantitative RT-PCR

After harvesting, the mouse skin samples were homogenized with a Bioprep-6 homogenizer (Allsheng, Hangzhou, China). Then, total RNA was isolated using Trizol reagent (Invitrogen) according to the manufacturer’s instructions. cDNA was synthesized from 5.0 µg of extracted total RNA using the First Strand cDNA Synthesis Kit (Thermo Fisher Scientific). Subsequently, the mRNA expression level was evaluated using the 7500 Real Time PCR system (Applied Biosystems, Foster City, CA, USA) and AccuPower^®^ 2X GreenStar™ qPCR Master Mix (Bioneer, Daejeon, Korea). The 2^-ΔΔCq^ method was used to determine the relative changes in the expression of each target gene. The expression of each gene was normalized to the expression of the *Gapdh* gene. The primer sequences for each gene are listed in **Supplementary Table 1**.

### Assessment of M1/M2 polarization

RAW 264.7 cells were seeded at a density of 1 × 10^6^ cells/well in 60-mm cell culture dishes. Cells were treated with vehicle (0.1% DMSO), lipopolysaccharide (LPS, 5 μg/mL) or LPS (5 μg/mL) + FABP4 inhibitor (0.1, 1 μM) for 24 h.

For evaluating M1/M2 polarization using qPCR, total RNA was isolated from the cell lysates using the Trizol reagent. For flow cytometry analysis, RAW 264.7 cells were stained with monoclonal antibodies against mouse CD206 (FITC, C068C2; BioLegend, San Diego, CA, USA) and CD11c (PerCP, N418, BioLegend). Cells were first gated based on forward and side scatter (FSC/SSC) to exclude debris. Doublets were excluded by FSC-A versus FSC-H gating. Viable cells were selected based on morphological parameters. CD11c^+^ and CD206^+^ populations were quantified within the singlet gate. Flow cytometric analyses were performed using a FACSymphony™ A1 Cell Analyzer (BD Biosciences). Data were analyzed using FlowJo software (BD Biosciences).

### Flow cytometric analysis of macrophage polarization

For flow cytometric analysis of mouse skin samples, ear skin tissues were harvested on day 6 and processed into single-cell suspensions. Briefly, the tissues were minced into small pieces and enzymatically digested with collagenase, DNase I, and trypsin to dissociate the skin tissue into single cells. The digested tissues were mechanically dissociated and sequentially filtered through 100-μm and 40-μm cell strainers to remove undigested tissue fragments and debris. Cells were then washed with PBS containing 2% fetal bovine serum.

Before antibody staining, cells were incubated with TruStain FcX™ PLUS anti-mouse CD16/32 antibody (clone S17011E; BioLegend, San Diego, CA, USA) to minimize nonspecific Fc receptor-mediated binding. Dead cells were excluded using Zombie Violet™ Fixable Viability Dye (BioLegend, San Diego, CA, USA), according to the manufacturer’s instructions. Cells were subsequently stained with fluorochrome-conjugated monoclonal antibodies against mouse CD45 APC/Cyanine7 (clone 30-F11; BioLegend), F4/80 APC (clone BM8; BioLegend), CD11c PerCP (clone N418; BioLegend), and CD206 FITC (clone C068C2; BioLegend).

To analyze macrophage polarization, cells were first gated based on forward scatter and side scatter to exclude debris, followed by doublet exclusion using FSC-A versus FSC-H. Viable cells were then selected by excluding Zombie Violet-positive dead cells. CD45^+^ immune cells were subsequently gated, and macrophages were identified as F4/80^+^ cells within the CD45^+^ population. CD11c^+^ and CD206^+^ populations were then quantified within the CD45^+^F4/80^+^ macrophage gate and interpreted as M1-associated/pro-inflammatory and M2-associated macrophage populations, respectively. Flow cytometric analyses were performed using a FACSymphony™ A1 Cell Analyzer, and data were analyzed using FlowJo software.

### Immunoblotting

Skin tissue samples and cultured cells, following treatment with the FABP4 inhibitor at the indicated concentrations, were homogenized and lysed in radioimmunoprecipitation assay (RIPA) buffer containing a protease (Roche, Basel, Switzerland) and phosphatase (GenDEPOT, Baker, TX, USA) inhibitor mixture. Total cell lysates and tissue extracts were separated by 10% sodium dodecyl sulfate polyacrylamide gel electrophoresis (SDS-PAGE) and transferred to polyvinylidene fluoride (PVDF) membranes. The membranes were blocked with 5% skim milk (Difco skim milk, 232100, BD Biosciences) in PBS containing 0.1% Tween 20 (PBST) for 1 h at 25 °C and probed with PPARG (1:1000 dilution, PA3-821A; Invitrogen), lamin B (1:1000 dilution, D4Q4Z; Cell Signaling Technology, Danvers, MA, USA);, and β-actin (1:1000 dilution, 13E5; Cell Signaling Technology) primary antibodies. Horseradish peroxidase-conjugated goat anti-rabbit antibody was used as a secondary antibody (1:10000, 32460; Invitrogen). The bands were visualized using the enhanced chemiluminescence detection system (Bio-Rad Laboratories, Hercules, CA, USA).

### Gene silencing via siRNA transfection

RAW 264.7 cells were seeded before transfection to reach approximately 50% confluence at the time of transfection. Cells were transfected with the negative control (scrambled) siRNA or FABP4-specific siRNA using Lipofectamine RNAiMAX(13778; Invitrogen). The control scrambled siRNA was obtained from Bioneer.

The siRNA sequences targeting FABP4 were as follows:

Forward 5’-CAU UGA ACU CUA CAA CAU U-3’;

Reverse 5’-AAU GUU GUA GAG GAG UUC AAU G-3’.

Briefly, siRNA and RNAiMAX were separately diluted in Opti-MEM (Invitrogen), combined, and incubated at room temperature for 20 min to allow complex formation. The siRNA-lipid complexes were then added to cells. After 6 hours of transfection, the culture medium was replaced and the cells were incubated at 37 °C in a CO_2_ incubator. After 24 hours, RAW 264.7 cells were lysed using TRIzol reagent (Invitrogen) according to the manufacturer’s instructions for subsequent RNA extraction.

### PPARG antagonism treatment

To pharmacologically antagonize PPARG, cells were seeded and cultured to reach approximately 80% confluence and treated with GW9662 (MedChemExpress, HY-16578R) or a FABP4 inhibitor. GW9662 was diluted in serum-free DMEM medium immediately before use. An equivalent volume of vehicle (DMSO) was added to the control group.

For experiments assessing the role of PPARG in macrophage polarization, GW9662 was co-administered with LPS and the FABP4 inhibitor, as specified in the figure legends. After 24 hours, cells were harvested and processed for qPCR analysis.

### Cell culture

RAW 264.7 cells were obtained from the Korean Cell Line Bank. The cells were cultured in Dulbecco’s modified Eagle medium (DMEM, Welgene, Gyeongsan, Korea) supplemented with 10% FBS (Welgene) and 1% penicillin (Gibco, Carlsbad, CA, USA) in a humidified incubator at 37 °C and 5% CO_2_.

### Statistical analysis

Statistical analyses were performed using GraphPad Prism (GraphPad Software, La Jolla, CA, USA). One-way analysis of variance (ANOVA), followed by Tukey’s *post hoc* tests, was used for comparing differences among three or more groups, whereas the Student *t*-test was used to compare differences between two groups. *P*-values *<* 0.05 were considered statistically significant; individual *P*-values (***P < 0.001, **P < 0.01, and *P < 0.05) are indicated in the figure legends.

## Results

### Single-cell RNA sequencing reveals macrophage polarization imbalance in psoriatic skin

**Figure 1:**
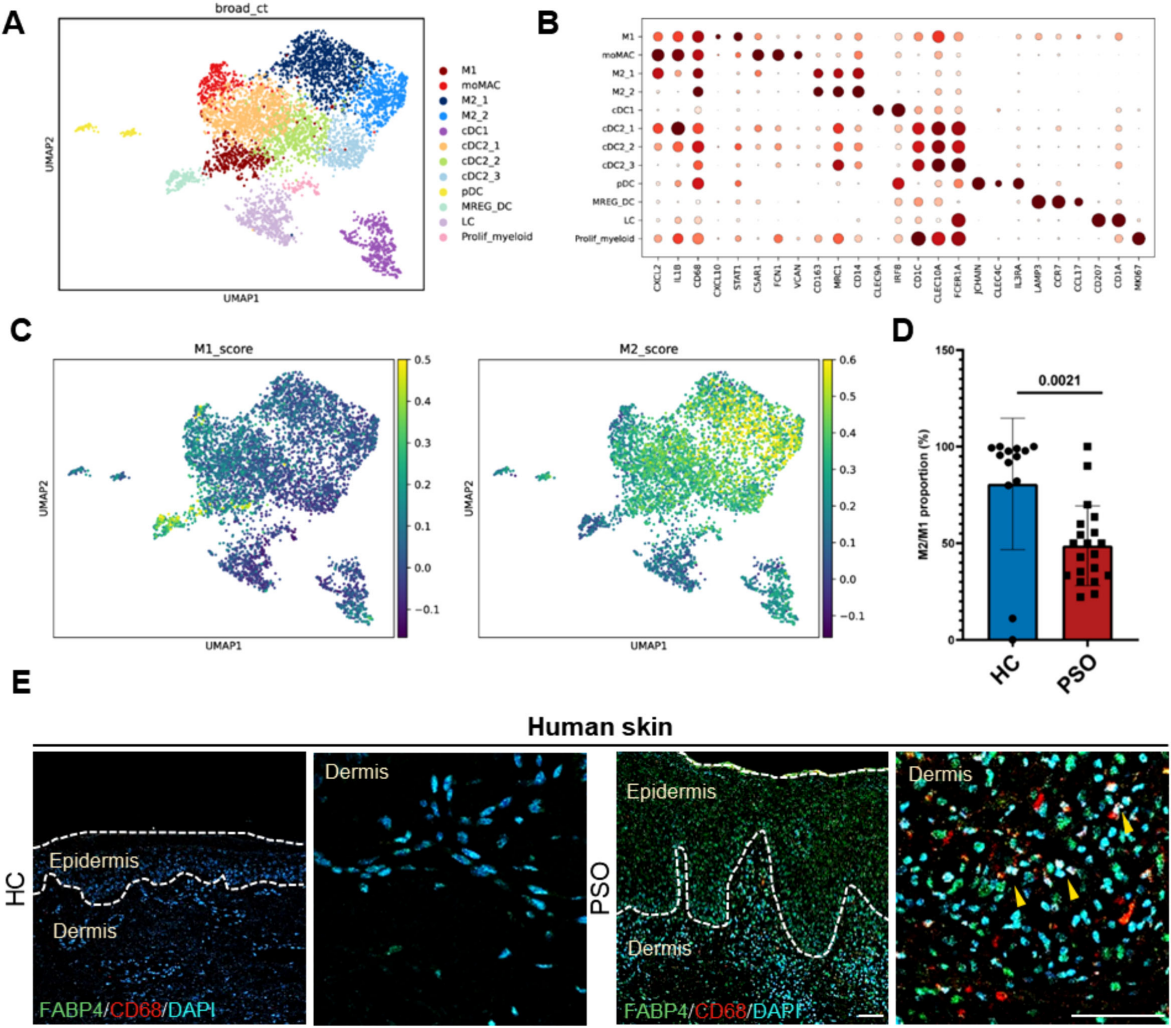
Macrophage polarization imbalance and FABP4 expression in psoriatic skin. **(A)** Uniform Manifold Approximation and Projection (UMAP) plot of skin myeloid cells grouped into 12 subclusters, each represented with a distinct color. **(B)** Dot plot showing the expression of representative marker genes for each myeloid subcluster. The color intensity reflects the average expression level, and dot size indicates the percentage of cells expressing each gene. **(C)** UMAP overlay of M1 and M2 macrophage signature genes in skin myeloid cells (26, 27). **(D)** Bar plot comparing the M2-to-M1 ratio between healthy controls (HC) and psoriasis patients (PSO). A significant decrease in the M2/M1 ratio was observed in PSO, indicating increased M1 polarization (p = 0.0021). **(E)** The representative immunofluorescence manifestations. Immunostaining analysis revealed distinct FABP4 localization predominantly in dermis of psoriasis skin. Magnified images of the indicated regions are shown. FABP4 is shown in green, CD68 in red, and nuclei are counterstained with DAPI in blue. Yellow arrowheads indicate FABP4^+^CD68^+^ macrophages in human psoriatic skin. n = 3 per group. FABP4, Fatty acid-binding protein 4; DAPI, 4′,6-diamidino-2-phenylindole; HC, Healthy Control; PSO, psoriasis.

While psoriasis involves a complex network of immune cells, macrophages act as a pivotal link between metabolic dysregulation and skin inflammation (21, 24). Notably, FABP4 is predominantly expressed in macrophages rather than T cells or B cells (21, 25), where it functions not only as a lipid chaperone but also as a driver of pro-inflammatory responses (15, 16). Given this specific link, we hypothesized that FABP4 in macrophages is a critical mediator of psoriatic inflammation. To establish the clinical relevance of this hypothesis and characterize the macrophage landscape in psoriatic skin, we performed single-cell RNA sequencing on dissociated surgical skin biopsies from patients with psoriasis (n = 7) using the 10X Genomics Chromium platform. The clinical characteristics of the in-house psoriasis cohort are summarized in **Supplementary Table 2**. For comparative analysis, we integrated our dataset with publicly available single-cell RNA-seq data from psoriatic (PSO) and healthy control (HC) skin (GSE153760, GSE158955, GSE173706, and GSE173205). In total, we analyzed samples from 20 patients with PSO and 15 HCs. After removing dead cells and doublets, 115238 cells were retained (n_PSO_ = 57 969; n_HC_ = 57 269), from which nine distinct cell clusters were identified (**Supplementary Figure 1A**). To dissect macrophage populations, we subsetted the myeloid cell compartment based on the expression of *AIF1*, *CD68*, and *CD1C* (**Supplementary Figure 1B**), resulting in 5131 cells (n_PSO_ = 3249; n_HC_ = 1882) (**Figure 1A**). Using well-known biomarkers, we further classified these cells into 12 subclusters: M1 (*CD68*^+^, *CXCL10*^+^, *STAT1*^+^); monocyte-derived macrophages (moMAC, *FCN1*^+^, *C5AR1*^+^, *VCAN*^+^); M2_1 and M2_2 (*CD163*^+^, *MRC1*^+^); cDC1 (*CLEC9A*^+^, *IRF8*^+^); cDC2_1, cDC2_2, and cDC2_3 (*CD1C*^+^, *CLEC10A*^+^); plasmacytoid dendritic cells (pDC, *CLEC4C*^+^, *IL3RA*^+^); MREG_DC (*LAMP3*^+^, *CCL17*^+^); Langerhans cells (LC, *CD207*^+^, *CD1A*^+^); and proliferating myeloid cells (Prolif_myeloid, *MKI67*^+^) (**Figure 1A**). We calculated M1 and M2 scores using established marker gene sets for macrophage polarization (26, 27). As shown in the UMAP visualization (**Figure 1C**), M1, moMAC, and MREG_DC subsets exhibited high M1 scores, whereas M2 scores were elevated across several subsets, with the highest levels observed in M2_1 and M2_2. To compare transcriptional features between polarized macrophage states, we examined marker expression patterns across the M1, M2_1 and M2_2 clusters (**Figure 1B**). This analysis revealed that the M1 cluster upregulated proinflammatory genes such as *IL1B, CXCL9, CXCL10, CCL20*, and *S100A9*, along with interferon-stimulated genes (ISGs) including *STAT1, IFI30*, and *ISG20*. In contrast, M2 subsets showed higher expression of canonical and tissue-remodeling-associated M2 genes such as *MRC1, CD163, CD209, MAFB, FOLR2*, and *CCL18* (**Supplementary Figure 1C**). To further assess macrophage polarization status, we compared the ratio of M2 (M2_1 and M2_2 clusters) to the M1 cluster between HCs and PSO samples. The M2/M1 proportion was significantly reduced in PSO skin compared with that in HCs (p = 0.0021) (**Figure 1D**), reflecting an altered balance in macrophage polarization states in the psoriatic microenvironment. Given the emerging evidence that dysregulated lipid metabolism is deeply intertwined with psoriatic inflammation (2, 6, 7), we hypothesized that altered intracellular lipid handling might drive this M1-skewed polarization. Specifically, we focused on FABP4, a major intracellular lipid chaperone in macrophages that has been implicated in promoting pro-inflammatory responses in other metabolic diseases (15, 17, 18).

### Elevated FABP4 expression in macrophages within psoriatic skin lesions of humans and mice

To determine whether FABP4 plays a role in the altered macrophage polarization observed in our scRNA-seq data, we investigated the expression of FABP4 in human skin from patients with psoriasis (**Figure 1E**). The results of immunofluorescence staining revealed multiple FABP4-positive cells scattered in the upper dermis of psoriatic skin lesions, which were not found in normal skin dermis. Given that FABP4 is predominantly expressed in monocytes and macrophages among immune cells (21, 24, 28), we stained the skin with anti-CD68 antibody and found multiple FABP4-positive macrophages in psoriatic skin. In the papillary dermis, FABP4-positive macrophages were present in the tortuous capillaries, which were extravasating from the capillaries and migrating into the dermis (**Supplementary Figure 2A**). In the reticular dermis, multiple scattered FABP4-positive macrophages were also present.

Next, we investigated FABP4 expression in the skin of imiquimod-induced psoriasis mice. Consistent with the results from human psoriatic samples, multiple FABP4-positive cells were present in the dermis of imiquimod-induced psoriasis mice (**Supplementary Figure 2B**). To further investigate the cell type expressing FABP4 in the dermis, we stained the skin with anti-F4/80 antibody and found that FABP4-positive cells also expressed F4/80, indicating that the FABP4-positive cells in skin lesions of imiquimod-induced psoriasis mice were macrophages (**Supplementary Figure 2C**). Compared with psoriatic lesions in mice, no FABP4-positive cells were present in the dermis of control mouse skin. FABP4 expression was significantly increased in psoriatic lesions of mouse skin compared with control mouse skin by approximately five-fold (**Supplementary Figure 2D**). These observations, combined with the known pro-inflammatory role of FABP4 in metabolic disorders, led us to investigate whether targeting FABP4 could serve as a viable therapeutic strategy to alleviate psoriatic inflammation by restoring the homeostatic balance of the skin microenvironment.

### Pharmacological inhibition of FABP4 ameliorates imiquimod-induced psoriasis-like skin inflammation

**Figure 2:**
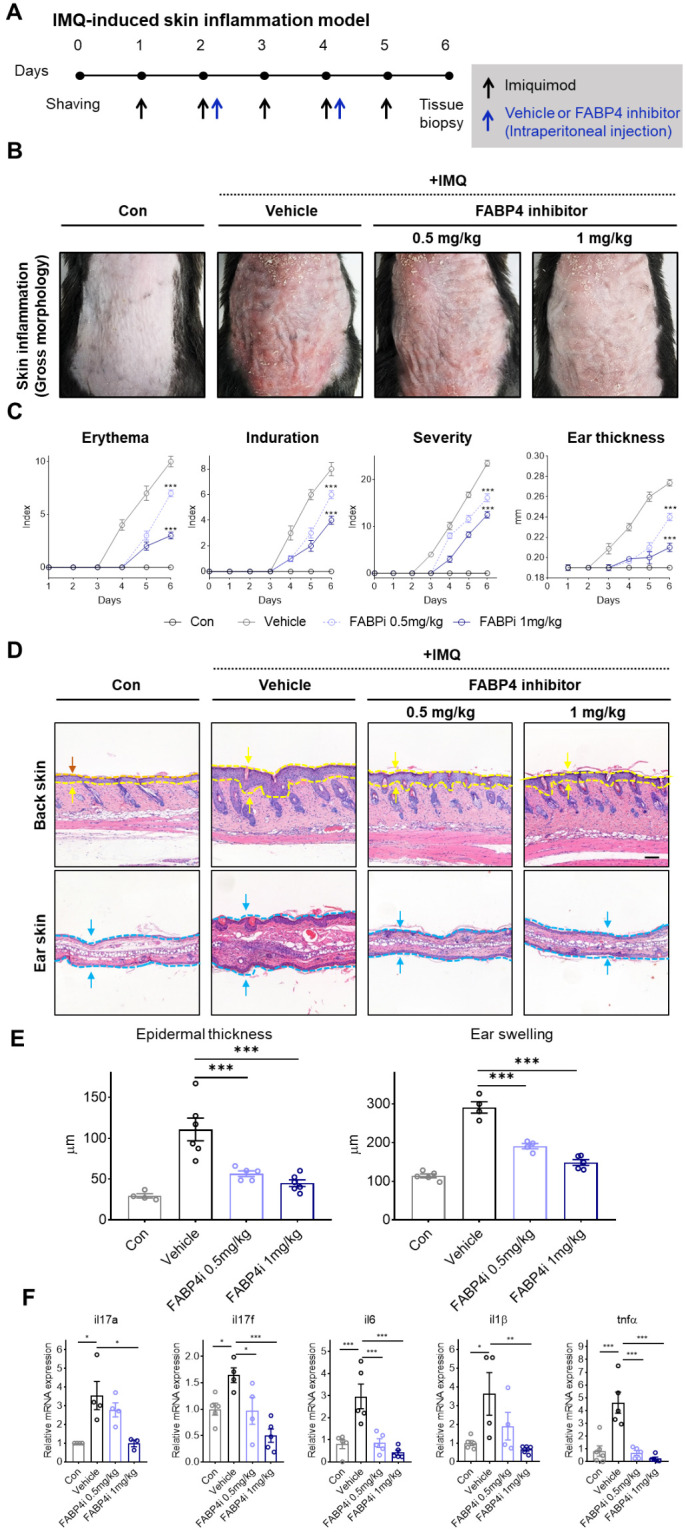
Inhibition of FABP4 attenuates imiquimod-induced psoriasiform skin inflammation *in vivo.* **(A)**. Experiment timeline. **(B)** The representative gross morphology of mice at day 6 (n=5 in each group). **(C)** The skin inflammation severity index (n=8 in each group). *p < 0.05, **p < 0.01, and ***p < 0.001 compared to the IMQ + Vehicle group for each index on day 6; two-way ANOVA with Tukey’s multiple comparisons test. **(D, E)** Representative histologic manifestations in back skin and ear skin (H&E staining). Epidermis was lined with yellow and blue dotted line. Epidermal thickening and ear swelling of mouse tissue (n=5 and n=6 spots in each group, respectively). *p < 0.05, **p < 0.01, and ***p < 0.001 compared to the IMQ + Vehicle group; one-way ANOVA with Tukey’s multiple comparisons test. Scale bar: 100 μm. **(F)** The relative gene expression levels of each inflammatory cytokine gene (n=4 or 5 in each group). IMQ: imiquimod, FABP4 inhibitor: Fatty acid-binding protein 4 inhibitor, il17a, interleukin-17a; il17f, interleukin-17f; il6, interleukin-6; il1β, interleukin-1 beta; tnfα, tumor necrosis factor-alpha.

Given the elevated expression of FABP4 in psoriatic macrophages and recent studies reporting that FABP4 inhibition can attenuate Th17-induced inflammation in nonobese diabetic and preeclampsia mice (19, 21), we investigated whether pharmacological inhibition of FABP4 could alleviate skin inflammation in the imiquimod-induced psoriasis model. First, we treated imiquimod-induced psoriasis mice with FABP4 inhibitor (BMS309403) and explored the effect of FABP4 inhibition on psoriasis-like skin inflammation (**Figure 2A**). As shown in **Figures 2B, C**, topical application of imiquimod induced characteristic psoriatic lesions, presenting with erythema, scaling, and induration. While the vehicle-treated group developed severe dermatitis and significant ear swelling, treatment with the FABP4 inhibitor significantly attenuated these pathological changes, with greater effects observed at the higher dose. The reductions in erythema, scaling, and ear thickness were statistically significant following FABP4 inhibition. Consistent with the macroscopic improvements, histological analysis revealed that FABP4 inhibition markedly suppressed epidermal hyperplasia and dermal inflammatory cell infiltration, which are hallmarks of psoriatic skin (**Figures 2D, E**). To confirm the molecular mechanism underlying these therapeutic effects, we evaluated changes in the gene expression of proinflammatory cytokines that play critical roles in psoriasis pathogenesis (**Figure 2F**). Our RT-qPCR analysis revealed that the expression levels of all investigated genes (*il17a, il17f, il6, il1β*, *and tnfα*) were significantly decreased in the FABP4 inhibitor-treated group, with greater suppressive effects observed at the higher dose. These results suggest that FABP4 inhibition effectively suppresses the inflammatory cascade central to psoriasis pathogenesis.

### FABP4 inhibition recalibrates the ratio between M1 and M2 macrophages in imiquimod-induced psoriasis mice

**Figure 3:**
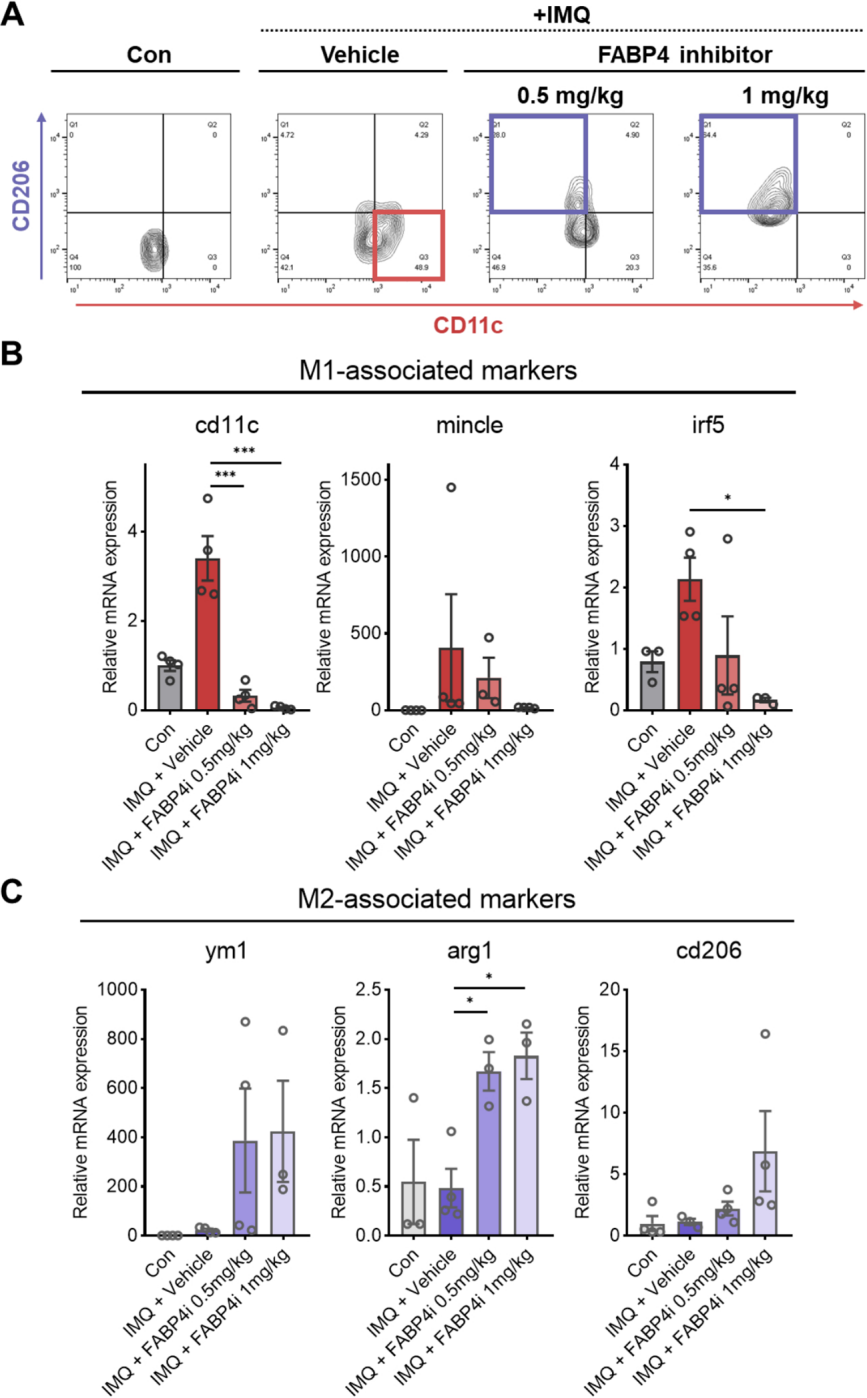
FABP4 inhibition modulates the macrophage polarization in imiquimod-induced psoriasiform mouse model. **(A)** Representative flow cytometry contour plots showing CD11c^+^ and CD206^+^ populations within viable CD45^+^F4/80^+^ macrophages isolated from mouse ear skin. CD11c^+^ and CD206^+^ cells were then quantified within the CD45^+^F4/80^+^ macrophage gate and interpreted as M1-associated/pro-inflammatory and M2-associated macrophage populations, respectively. Quantification was performed using samples from each group (n = 4 per group). **(B)** Quantitative RT-PCR analysis of M1 macrophage markers (cd11c, mincle and irf5) showing dose-dependent suppression by FABP4 inhibitor treatment. **(C)** Expression levels of M2 macrophage markers (ym1, arg1 and CD206) demonstrating enhanced M2 polarization following FABP4 inhibition. Data are presented as mean ± SE; *P < 0.05, and ***P < 0.001 compared to the Vehicle. Data were statistically analyzed by one-way ANOVA. FABP4i: Fatty acid-binding protein 4 inhibitor, Mincle: macrophage-inducible C-type lectin, irf5: interferon regulatory factor 5, arg1: arginase 1.

Our immunofluorescence analysis suggested that cells expressing FABP4 were mainly macrophages in the dermal skin of imiquimod-induced psoriasis mice. In parallel, single-cell RNA sequencing revealed an altered macrophage polarization state skewed toward the M1 phenotype within the psoriatic microenvironment. These findings led us to hypothesize that FABP4 inhibition might alleviate psoriatic inflammation by restoring the balance of macrophage polarization. To test this, we investigated the effect of FABP4 inhibitors on macrophage polarization in imiquimod-induced psoriasis mice. As shown in **Figure 3A**, flow cytometric analysis revealed that imiquimod application induced M1 polarization, characterized by an increased proportion of CD11c-expressing proinflammatory macrophages. However, FABP4 inhibition significantly mitigated this skewing; the treatment reduced M1 abundance while concurrently increasing the population of CD206-expressing M2 macrophages. Representative dot plots for the flow cytometric analysis are provided in **Supplementary Figure 5**, showing that CD11c^+^ and CD206^+^ populations were quantified within the viable CD45^+^F4/80^+^ macrophage gate using identical display settings across groups. We further validated these findings by analyzing gene expression in mouse skin tissue lysates. Consistent with the flow cytometry results, FABP4 inhibition significantly reduced the expression of M1 signature genes (*Cd11c, Mincle*, and *Irf5*) (**Figure 3B**). Conversely, we observed a concentration-dependent upregulation of M2 macrophage-associated markers (*Ym1, Arg1*, and *Cd206*) following FABP4 inhibitor treatment (**Figure 3C**). Collectively, these *in vivo* results suggest that FABP4 inhibition alleviates psoriatic inflammation by shifting the macrophage population from a proinflammatory M1 phenotype toward an anti-inflammatory M2 phenotype.

### FABP4 inhibition directly modulates macrophage polarization *in vitro*

**Figure 4:**
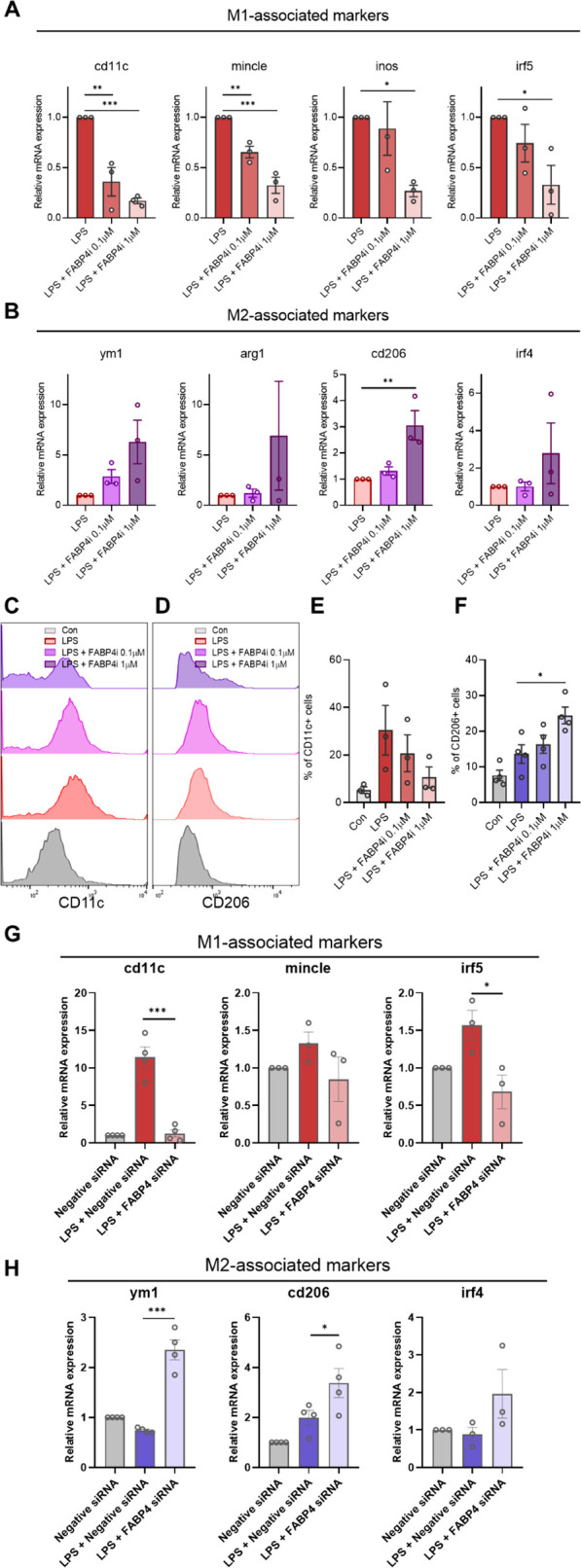
FABP4 inhibition regulated macrophage polarization in LPS-stimulated macrophages. **(A, B)** RAW 264.7 macrophages were stimulated with LPS and treated with the FABP4 inhibitor (0.1, 1μM). Quantitative RT-PCR analysis was performed to assess expression of M1-associated genes (cd11c, mincle, iNOS, and irf5) and M2-associated genes (ym1, arg1, cd206 and irf4). **(C, D)** Representative flow cytometry histograms showing surface expression of CD11c and CD206 in RAW 264.7 macrophages under LPS and FABP4 inhibitor. **(E, F)** Quantification of CD11c^+^ and CD206^+^ macrophage populations from three independent experiments. FABP4 inhibition significantly decreased the proportion of CD11c^+^ cells while increasing CD206^+^ cells compared with LPS treatment alone. **(G)** RAW 264.7 macrophages were transfected with control or FABP4-specific siRNA and stimulated with LPS. Quantitative RT–PCR analysis of M1-associated genes (*Cd11c, Mincle*, and *Irf5*) demonstrated that FABP4 silencing significantly suppressed LPS-induced pro-inflammatory gene expression. **(H)** Under the same conditions, quantitative RT–PCR analysis of M2-associated genes (*Ym1, Cd206*, and *Irf4*) showed enhanced alternative macrophage polarization following FABP4 knockdown. Data are presented as mean ± SE; *P < 0.05, **P < 0.01, and ***P < 0.001 compared to the Vehicle. Data were statistically analyzed by one-way ANOVA.

To corroborate these *in vivo* findings and determine if FABP4 inhibition directly regulates macrophage plasticity independent of the tissue microenvironment, we performed experiments using the RAW 264.7 macrophage cell line. Cells were stimulated with LPS to induce an inflammatory state in the presence or absence of the FABP4 inhibitor (BMS309403) for 24 h. To exclude the possibility that the observed effects were due to cytotoxicity, we performed a CCK-8 cell viability assay and confirmed that FABP4 inhibitor treatment did not significantly affect RAW 264.7 cell viability at concentrations up to 10 μM (**Supplementary Figure 4**). Consistent with the results from the psoriasis mouse model, FABP4 inhibition induced a recalibration of macrophage polarization in a concentration-dependent manner. The expression of M1 macrophage markers, including *Cd11c, Mincle* (macrophage-inducible C-type lectin), *iNOS* (inducible nitric oxide synthase), and *Irf5* (interferon regulatory factor 5), was dramatically downregulated following FABP4 inhibition (**Figure 4A**). Conversely, FABP4 inhibition promoted the expression of M2 markers, indicating polarization toward the alternatively activated phenotype; *Ym1* (chitinase 3-like 3), *Arg1* (arginase 1), *Cd206*, and *Irf4* (interferon regulatory factor 4) showed increased expression in response to FABP4 inhibition (**Figure 4B**). We further confirmed these phenotypic changes using flow cytometry. The percentage of CD11c-positive cells (M1) decreased following FABP4 inhibition (**Figures 4C, E**). More notably, the population of CD206-positive cells increased significantly following treatment with FABP4 inhibitor at 1 μM (P < 0.05) compared with that in the LPS-treated group (**Figures 4D, F**). To further validate the target specificity of FABP4 inhibition, we performed gene silencing experiments using small interfering RNA (siRNA) targeting *Fabp4*. Consistent with the pharmacological inhibition results, *Fabp4* knockdown in LPS-stimulated macrophages significantly downregulated M1 markers while concurrently upregulating M2 markers (*Arg1, Ym1, Cd206*), confirming that the observed polarization shift is specifically mediated by FABP4 (**Figures 4G, H**). These results demonstrated that FABP4 inhibition effectively modulates macrophage polarization by suppressing proinflammatory M1 markers while enhancing anti-inflammatory M2 markers.

### PPARG regulon activity is intrinsically linked to M2 macrophage polarization in psoriatic skin

**Figure 5:**
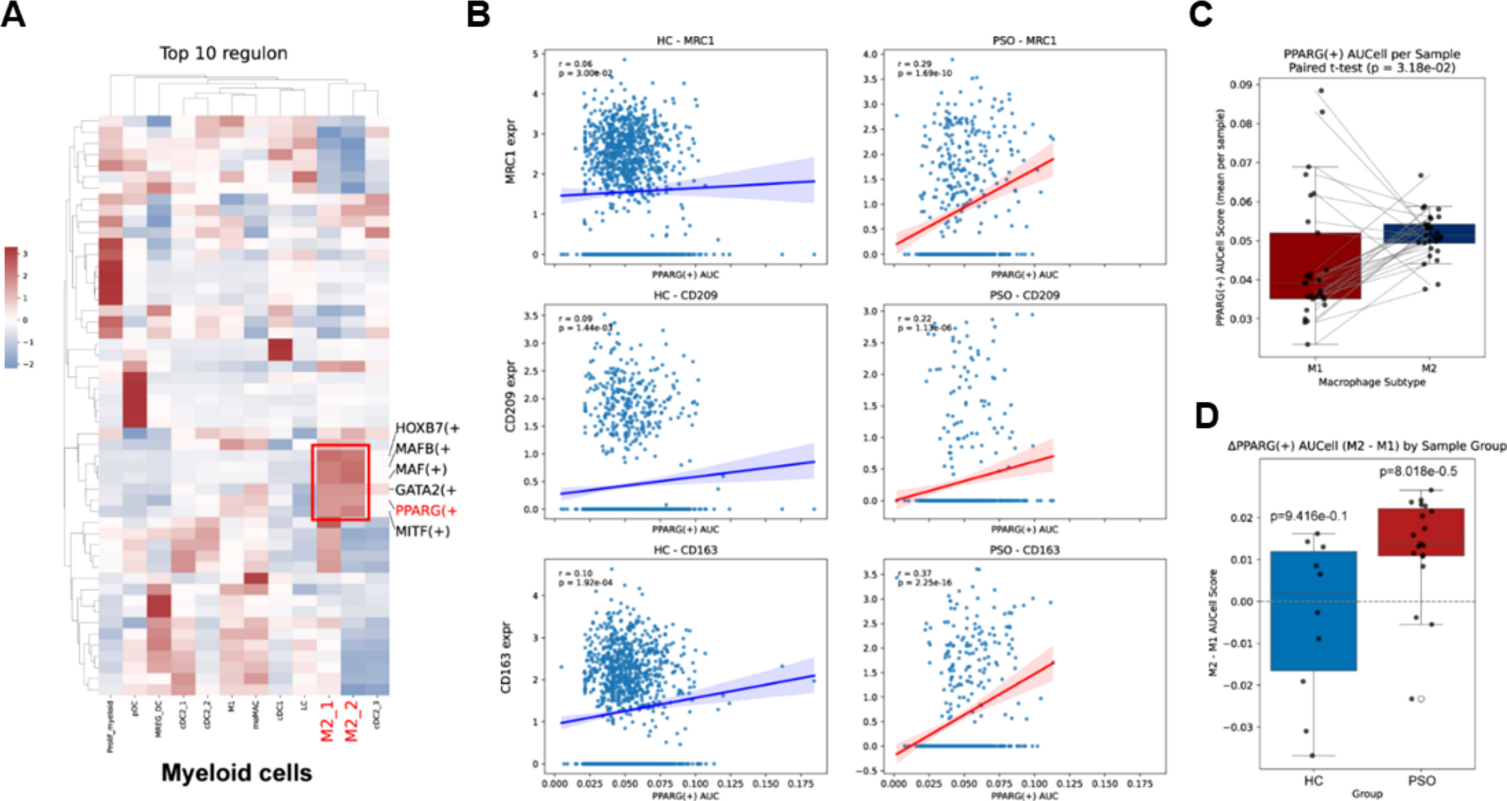
PPARG regulon activity analysis reveals altered macrophage polarization patterns in psoriatic skin. **(A)** Heatmap showing the relative activity of the top 10 differentially expressed regulons (DERs) across myeloid subpopulations in human skin scRNA-seq data, identified by SCENIC analysis. Columns represent myeloid clusters and rows represent regulons. Regulons with increased activity in both M2_1 and M2_2 macrophage clusters, including HOXB7(+), MAFB(+), MAF(+), GATA2(+), PPARG(+), and MITF(+), are highlighted in a red box on the heatmap. **(B)** Scatter plots showing correlations between PPARG regulon activity (x-axis) and expression of M2-associated genes (MRC1, CD209, and CD163; y-axis) in healthy control (HC, left) and psoriasis (PSO, right) samples. Each dot represents a single cell. The solid line indicates the linear regression fit, and the shaded area shows the 95% confidence interval. **(C)** Box plot comparing PPARG(+) AUCell scores between M1 and M2 macrophages across all samples. Each dot indicates the average score per sample. Lines connect paired values from the same sample, and comparison was performed using a paired t-test (p = 0.0318). **(D)** Comparison of ΔPPARG(+) activity (M2 – M1 AUCell score) between healthy controls (HC) and psoriasis patients (PSO). Paired t-tests were conducted separately within each group. No significant difference was observed in HC (left), whereas PSO samples (right) showed a significant increase in PPARG(+) activity in M2 compared to M1 (p = 8.018e-05), indicating that PPARG activation in M2 macrophages may be enhanced specifically under psoriatic conditions.

To elucidate the molecular mechanism by which FABP4 regulates macrophage polarization, we sought to identify upstream transcription factors (TFs) driving this process. We applied the SCENIC (Single-Cell rEgulatory Network Inference and Clustering) pipeline to infer TF regulon activity. Based on the single-cell dataset generated in this study, we calculated the top 10 regulons for each myeloid cell cluster (**Figure 5A**). A heatmap was generated with hierarchical clustering applied to both rows and columns, which revealed that the M2_1 and M2_2 clusters shared a similar regulon profile. Among the top regulons, six transcription factors (*HOXB7*^+^, *MAFB*^+^, *MAF*^+^, *GATA2*^+^, *PPARG^+^*, and *MITF*^+^) showed commonly increased activity in the M2_1 and M2_2 clusters. *MAFB*^+^ and *MAF*^+^ transcription factors have been reported to drive anti-inflammatory M2 macrophage polarization (29, 30). PPARG^+^ is induced upon IL-4 and IL-13 stimulation and contributes to the downregulation of M1-associated genes, in part by interfering with the activity of transcription factors such as AP-1, STAT, and NF-κB (29, 30). We selected one of the six transcription factors identified in our analysis, PPARG, for further investigation of the underlying mechanism, based on the previously reported inverse relationship between FABP4 and PPARG where FABP4 triggers the ubiquitination and proteasomal degradation of PPAR (31). To further explore the functional relevance of PPARG^+^ activity in M2 polarization, we assessed its correlation with the expression of key M2 markers. We performed Spearman correlation analysis between PPARG^+^ AUC scores and the expression levels of *MRC1, CD209, and CD163* within macrophage populations from both HC and PSO samples (**Figure 5B**). In PSO skin, PPARG^+^ activity was significantly correlated with increased expression of all three M2 markers (MRC1: r = 0.29, p = 1.18e^-10^; CD209: r = 0.22, p = 1.13e^-06^; CD163: r = 0.37, p = 2.27e^-16^). In contrast, we observed only weak or marginal correlations in HC samples, suggesting that the regulatory influence of PPARG^+^ on M2 marker expression may be more prominent in psoriatic lesions. We next compared PPARG^+^ regulon activity between M1 and M2 macrophage subsets. Paired analysis revealed that PPARG^+^ AUC scores were significantly higher in M2 compared with those in M1 macrophages (p = 3.18e^-02^) (**Figure 5C**). Subsequently, we evaluated whether this pattern of PPARG^+^ activation differed across disease and control conditions. We performed paired *t*-tests separately within the HC and PSO groups (**Figure 5D**). In HC samples, the difference was not significant (p = 0.9416), whereas PSO samples exhibited a significantly greater ΔPPARG score (p = 8.018e^-05^), suggesting that PPARG-driven M2 polarization is selectively enhanced in psoriatic skin. These findings suggest that PPAR signaling is a critical determinant of M2 polarization specifically in the context of psoriatic inflammation.

### FABP4 inhibition restored PPARG protein expression and activity suppressed by imiquimod-induced inflammation in psoriasis-like mouse model

**Figure 6:**
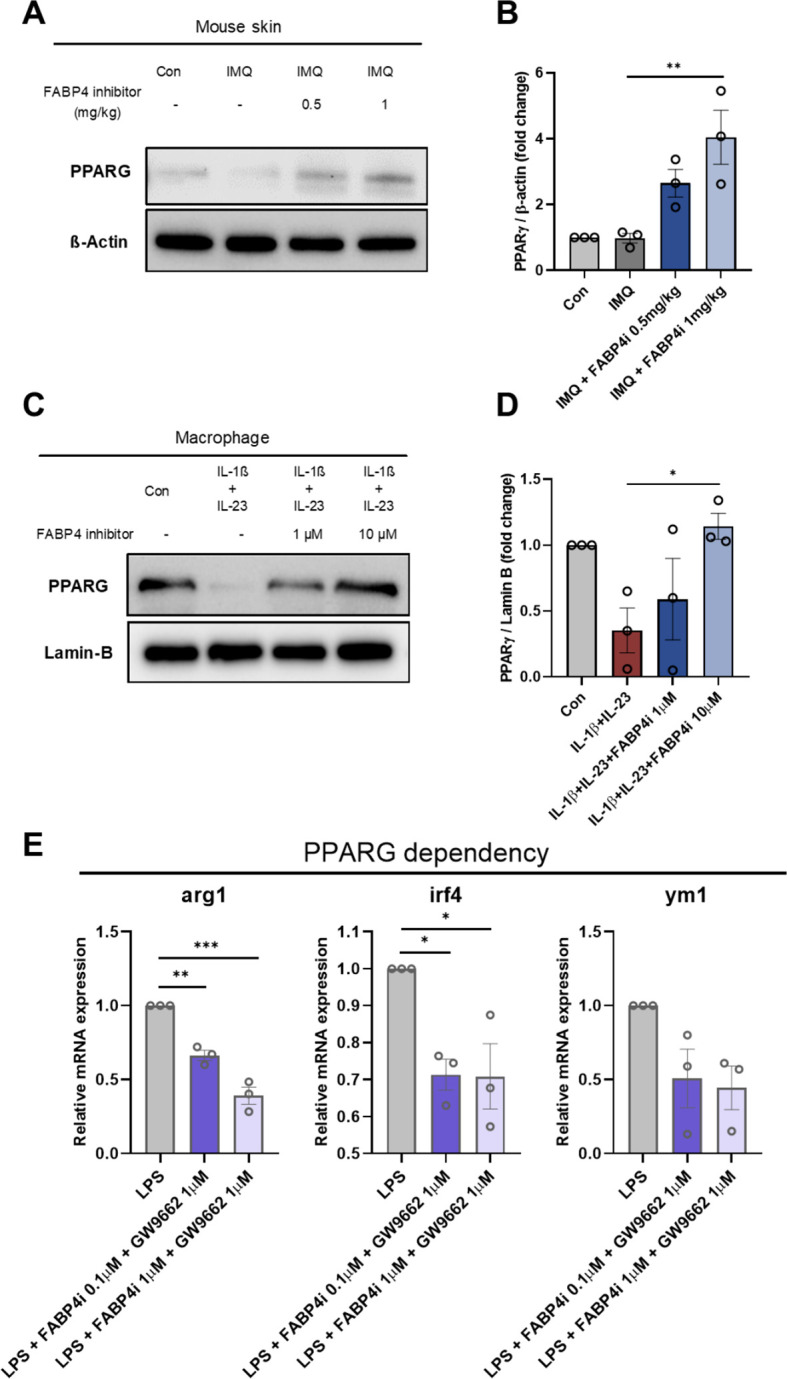
FABP4 regulates macrophage polarization through a PPARG-dependent mechanism. **(A)** Immunoblot analysis of PPARG protein expression in back skin tissues from imiquimod (IMQ)-treated mice administered vehicle or FABP4 inhibitor as indicated. β-Actin served as a loading control. **(B)** Densitometric quantification of PPARG protein levels in mouse skin tissue normalized to β-Actin. **(C)** Macrophages were exposed to an inflammatory cytokine milieu (IL-1β, IL-18 and IL-23) in the presence or absence of the FABP4 inhibitor, followed by immunoblot analysis of PPARG expression. Lamin B was used as a loading control. **(D)** Quantification of PPARG protein levels normalized to Lamin B from the immunoblot. **(E)** RAW 264.7 macrophages were stimulated with LPS and treated with a FABP4 inhibitor (0.1 μM or 1 μM) in the presence or absence of the selective PPARG antagonist GW9662, as indicated. After 24 h, mRNA expression levels of M2-associated genes (Arg1, Irf4, and Ym1) were analyzed by quantitative RT–PCR. Data are presented as mean ± SE; *P < 0.05, **P < 0.01, and ***P < 0.001 compared to the Vehicle. Data were statistically analyzed by one-way ANOVA.

Based on the PPARG regulon activity analysis results suggesting a correlation between PPARG activity and M2 macrophages in psoriatic skin lesions and the results from previous studies showing that FABP4 promoted PPARG ubiquitination, thereby inducing its degradation (31), we postulated that FABP4 influences macrophage polarization by modulating PPARG and conducted further investigations into the mechanisms underlying macrophage polarization modulation by FABP4 in the imiquimod-induced psoriasis mouse model. As shown in **Figures 6A, B**, topical imiquimod application resulted in PPARG reduction in imiquimod-induced psoriasis mice. Conversely, FABP4 inhibition demonstrated a tendency to reverse these alterations. In addition, FABP4 inhibition also restored PPARG activity, which was suppressed by topical imiquimod application (**Supplementary Figure 3**). We made similar observations in macrophages stimulated with IL-1β and IL-23 *in vitro*: IL-1β and IL-23 treatment decreased the PPARG protein levels, whereas FABP4 inhibition again restored this change (**Figures 6C, D**; **Supplementary Figure 4**). Collectively, these results suggest a potential link between FABP4 inhibition, restoration of PPARG activity, and the promotion of anti-inflammatory M2 macrophage polarization.

### FABP4 inhibition restores PPARG protein stability and activity to drive M2 polarization

**Figure 7:**
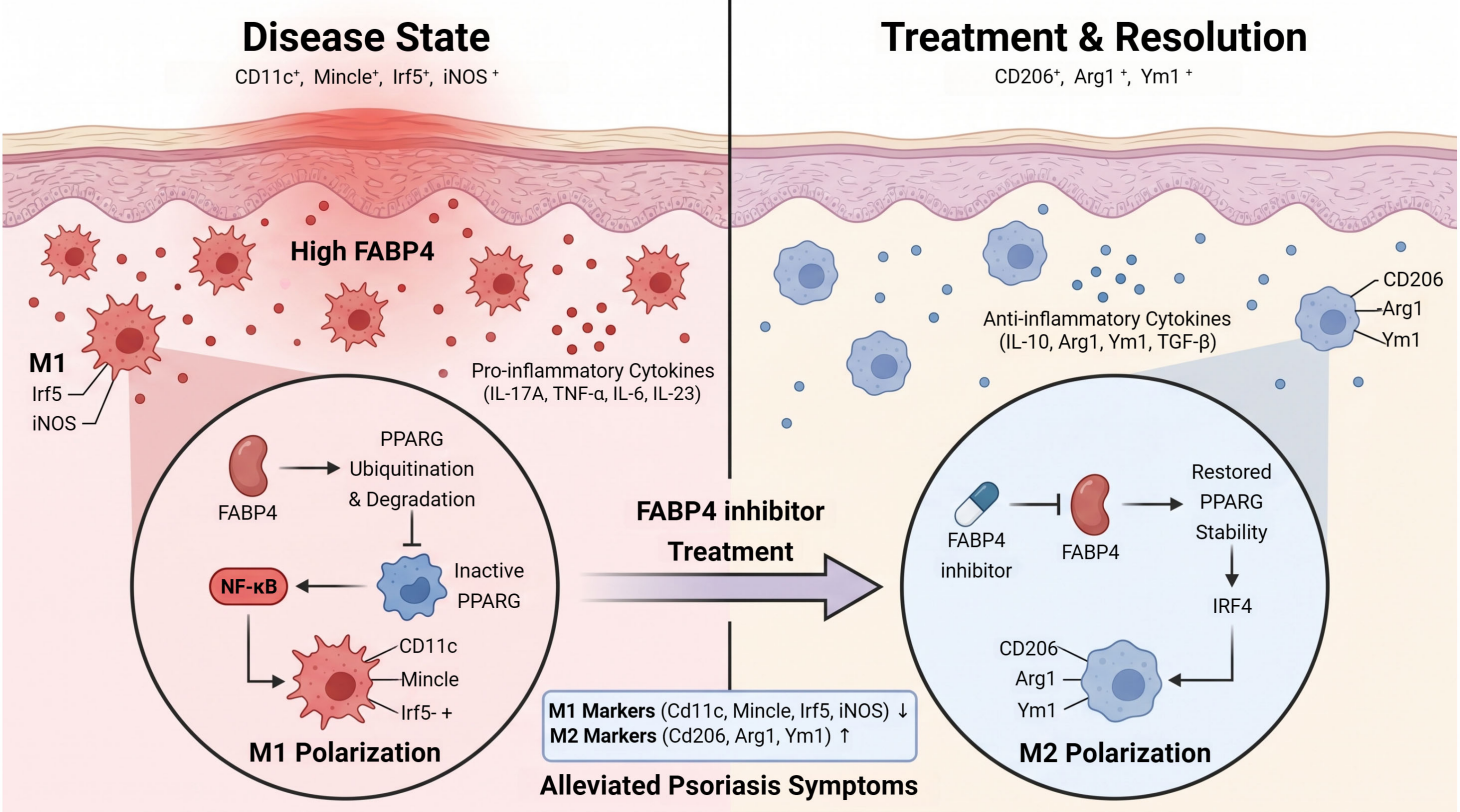
Schematic illustration of FABP4-mediated immune modulation in psoriasis skin inflammation. In psoriatic skin, elevated FABP4 expression in infiltrating macrophages promotes proinflammatory M1 polarization and suppresses PPARG activity. Pharmacological inhibition of FABP4 restores PPARG protein stability and activity, thereby shifting macrophage polarization toward an anti-inflammatory M2 phenotype. The increased expression of M2-associated markers (Arg1, CD206, Irf4) promotes the resolution of inflammation and alleviates psoriasis-like skin pathology. This schematic summarizes the FABP4–PPARG axis as a key regulatory pathway controlling macrophage polarization in psoriatic inflammation.

Building upon our SCENIC analysis which identified a strong correlation between PPAR regulon activity and M2 macrophage polarization in psoriatic lesions and our results showing the potential link between FABP4 inhibition, restoration of PPARG activity, and the promotion of anti-inflammatory M2 macrophage polarization, we sought to determine if FABP4 directly regulates PPAR stability in this context. Given previous studies demonstrating that FABP4 promotes PPAR ubiquitination and subsequent proteasomal degradation (29), we hypothesized that the elevated FABP4 in psoriatic skin drives inflammation by suppressing PPAR. Finally, to prove that FABP4 regulates macrophage polarization specifically through a PPAR-dependent mechanism, we utilized the selective PPARG antagonist, GW9662. While FABP4 inhibition successfully induced the expression of M2 markers (*Arg1, Irf4*), this induction was effectively abrogated by co-treatment with GW9662 (**Figure 6E**). Collectively, these results provide compelling evidence that FABP4 inhibition alleviates psoriatic inflammation by restoring PPARG protein expression and driving anti-inflammatory M2 macrophage polarization. Finally, we summarized the proposed mechanism of FABP4-mediated macrophage polarization in psoriasis (**Figure 7**).

## Discussion

Previous studies have revealed the association between FABP4 and psoriasis (32). Serum FABP4 levels are increased in patients with psoriasis and correlate with psoriasis comorbidities (23, 32). However, the specific role of FABP4 in psoriasis pathogenesis remains not well studied. In this study, utilizing single-cell RNA sequencing analysis, we identified a distinct macrophage polarization imbalance characterized by M1 skewing in psoriatic skin lesions, and identified PPARG as a master transcription factor driving the anti-inflammatory M2 phenotype. Given that FABP4 was highly expressed in infiltrating macrophages in human and mouse skin of psoriasis and is known to promote PPARG degradation, we investigated their interplay in the pathogenesis of psoriasis. Using an imiquimod-induced psoriasis mouse model, we demonstrated that FABP4 inhibition ameliorated skin inflammation by recalibrating macrophage polarization towards an anti-inflammatory M2 phenotype. Furthermore, using siRNA-mediated knockdown and the PPARG antagonist, we found that FABP4 inhibition exerts its therapeutic effects specifically by restoring PPARG protein, thereby driving M2 polarization as a primary mechanism for alleviating psoriatic inflammation. Collectively, these findings elucidate a novel relationship among FABP4, PPARG, and macrophage polarization in the pathogenesis of psoriasis.

Macrophages are innate immune cells derived from circulating monocytes (33). The M1/M2 classification used in this study should be interpreted as a simplified functional framework, as macrophage activation in psoriasis represents a spectrum of heterogeneous and dynamic states. When macrophages are stimulated by signals from the surrounding microenvironment, they activate and differentiate into two phenotypes: proinflammatory M1 and immune modulatory M2 macrophages (33, 34). First, we analyzed single-cell RNA sequencing data from psoriatic skin lesions and investigated macrophage characteristics in patients with psoriasis. Because clinical metadata were not consistently available for the integrated public datasets, the human scRNA-seq analysis was interpreted primarily in the context of psoriatic versus healthy skin differences rather than disease severity-associated variation across the full cohort. Our analysis showed that macrophages from psoriatic skin showed an altered balance in macrophage polarization toward M1, showing upregulated proinflammatory gene expression. To further study the mechanistic role of FABP4 in psoriasis pathogenesis, we explored the role of FABP4 in macrophage polarization in imiquimod-induced psoriasis mice. Our study found that imiquimod application increased the number of CD11c-expressing M1 macrophages, whereas FABP4 inhibition recalibrated macrophage polarization by decreasing M1 abundance while increasing the number of CD206-expressing M2 macrophages. These results suggested that FABP4 plays a role in macrophage activation and polarization toward M1 in the pathogenesis of imiquimod-induced psoriasis mice, implying that controlling FABP4 can alleviate proinflammatory changes in macrophages in imiquimod-induced psoriasis mice. These findings demonstrate that FABP4, whose expression is elevated in psoriasis, contributes to disease pathogenesis by modulating macrophage polarization, thereby confirming its mechanistic role in psoriasis development. However, the IMQ-induced psoriasis model primarily reflects an acute inflammatory response rather than the chronic and relapsing nature of human psoriasis. Accordingly, further studies using chronic or therapeutic treatment models are needed to evaluate the translational potential of FABP4 inhibition. Although LPS stimulation is widely used to model pro-inflammatory macrophage activation, it does not fully reflect the complex cytokine milieu and multicellular interactions characteristic of psoriatic inflammation.

To further investigate the mechanism by which FABP4 regulates macrophage polarization, we applied the SCENIC pipeline to infer transcription factor activity. Our analysis identified PPARG as a top-ranking transcription factor driving the M2 macrophage program in psoriatic skin. PPARG activity was significantly correlated with increased expression of M2 markers such as MRC1, CD209, and CD163 in psoriatic skin, in line with previous studies revealing the role of PPARG in macrophage polarization toward the immune modulating M2 phenotype (35–38). Notably, while overall PPARG protein levels are suppressed in the whole psoriatic tissue, high PPARG regulon activity is uniquely observed in the M2 macrophage subsets. Mechanistically, FABP4 has been previously reported to function as a negative regulator of PPARG by triggering its ubiquitination and subsequent proteasomal degradation in adipocytes (31). Based on our TF regulon activity results and previous studies revealing the association between FABP4 and PPARG activity, we hypothesized that FABP4 controls macrophage polarization via regulating PPARG activity and investigated the relationship between FABP4 and PPARG in imiquimod-induced psoriasis mice. Our study found that FABP4 inhibition restored PPARG protein level and activity, which were decreased by imiquimod-induced psoriasis-like inflammation in imiquimod-induced mice. In addition, our *in vitro* experiment showed that FABP4 inhibition increased the PPARG protein level in macrophages, which was decreased by IL-1β and IL-23 stimulation. Collectively, these findings suggest that FABP4 controls macrophage plasticity in psoriasis by regulating the turnover of PPARG, and its inhibition releases this brake to promote the resolution of inflammation.

Macrophage polarization is closely linked to cellular metabolic reprogramming, and PPARG is a key regulator connecting lipid metabolism with macrophage polarization. Given that FABP4 functions as an intracellular lipid chaperone and that FABP4 inhibition restored PPARG expression in our study, the FABP4–PPARG axis may also influence metabolic remodeling during macrophage polarization. Further investigation of FAO-related enzymes is needed to better define the metabolic effects of FABP4 inhibition in macrophages during psoriatic inflammation.

To rigorously verify whether the observed therapeutic effects were specifically mediated through the FABP4–PPAR axis, we conducted mechanistic validation using both genetic and pharmacological approaches. The knockdown of *Fabp4* via siRNA in macrophages reproduced the M2-polarizing phenotype characterized by the upregulation of *Arg1* and *Cd206*, which mirrored the effects of pharmacological FABP4 inhibition. This genetic confirmation effectively excludes potential off-target effects of the inhibitor. Moreover, the abrogation of FABP4 inhibitor–induced M2 polarization by the selective PPARG antagonist GW9662 further substantiates that the anti-inflammatory effects of FABP4 inhibition are dependent on restoration of PPARG signaling, confirming that FABP4 acts as an upstream suppressor of this pathway. Collectively, these findings provide mechanistic evidence that suppression of FABP4 restores PPARG stability and activity, which in turn drives macrophage reprogramming toward a pro-resolving M2 phenotype.

Recent studies have found that M1 macrophages play important roles in the early phase of psoriasis: M1 macrophages produce IL-23, which plays a critical role in Th17 cell differentiation in psoriasis (39–42). Based on the results, we speculated that FABP4 can be a critical regulator in psoriasis pathogenesis by regulating macrophage polarization. Our findings on the relationship between FABP4 and macrophage polarization in psoriasis may also provide new insights into the mechanisms underlying psoriasis-associated comorbidities: FABP4-driven activation of M1 macrophages may contribute to the development of insulin resistance, potentially linking FABP4 to the broader spectrum of metabolic diseases observed in patients with psoriasis (1, 43). However, notably, adipocytes, which express FABP4, have also been implicated in psoriasis pathogenesis (31, 44). This raises the possibility that FABP4 inhibition could modulate psoriatic inflammation via alterations in adipocyte function. Therefore, further investigations into adipocyte-mediated mechanisms of FABP4 action are warranted.

In summary, our study demonstrates that FABP4 plays a critical role in psoriatic skin inflammation by modulating macrophage polarization through the regulation of PPARG. We showed that FABP4 inhibition suppresses pro-inflammatory M1 polarization while promoting the shift toward the anti-inflammatory M2 phenotype, thereby effectively alleviating psoriasis-like skin inflammation *in vivo*. Mechanistically, we identified a direct link between FABP4 and PPARG, where FABP4 inhibition acts upstream to restore endogenous PPARG that is otherwise suppressed in the inflamed tissue context. While direct PPARG agonists have shown potential in psoriasis, their clinical utility is often limited by systemic side effects (45). Our findings suggest that targeting FABP4 offers a strategic advantage by enhancing physiological PPARG signaling specifically within the inflammatory microenvironment, potentially avoiding the adverse effects associated with systemic PPARG hyperactivation. Collectively, these results provide compelling evidence that FABP4 serves as a master regulator of psoriatic inflammation by orchestrating macrophage polarization, positioning it as a promising therapeutic target for psoriasis and other macrophage-driven inflammatory skin disorders.

## Data Availability

The original contributions presented in the study are publicly available. This data can be found here: GEO, accession number GSE337821.
