# Supplementary material for: Inhibition of fatty acid-binding protein 4 alleviates psoriasis-like skin inflammation by modulating macrophage polarization
Source: Front Immunol. 2026 Aug 3;17:1830077. doi: 10.3389/fimmu.2026.1830077 (PMC13478135; doi:10.3389/fimmu.2026.1830077)
Supplement: Supplementary file 1 [file DataSheet1.docx]

**Supplementary materials and methods**

**Human samples**

Human samples were obtained under Institutional Review Board (IRB) approval from two institutions. Psoriasis lesions (Pso#1–3) were collected from the Department of Dermatology, Severance Hospital, Cutaneous Biology Research Institute, Yonsei University College of Medicine, Republic of Korea (IRB No. CR324032), and additional samples (Pso#4–7) were obtained from the Department of Dermatology, Hanyang University College of Medicine, Republic of Korea (IRB No. HYUH 2017-10-015). All samples were collected via 2–4 mm punch biopsy and were immediately transported to Seoul National University College of Medicine, Republic of Korea. Each biopsy was divided into two halves: one half was enzymatically dissociated into a single-cell suspension for single-cell RNA sequencing (scRNA-seq), whereas the other half was fixed in formalin for subsequent immunohistochemical analysis.

**Peripheral blood mononuclear cell (PBMC) isolation**

At the time of skin biopsy, paired peripheral blood samples were collected from the same donors and processed using BD Vacutainer® CPT™ Cell Preparation Tubes (BD Biosciences) according to the manufacturer’s protocol. Samples were centrifuged at 1800 × g for 20 min at 25 °C, and the mononuclear cell layer was isolated and washed with RPMI-1640 medium supplemented with 10% fetal bovine serum (FBS). Dissociated PBMCs were resuspended in freezing medium, transferred into cryovials, and stored at −80 °C for long-term preservation.

**Single-cell preparation**

For single-cell preparation, skin tissues were finely minced and digested with freshly thawed 2 mg/mL cold Liberase TL (Research Grade, 05401020001, Roche) at 37 °C for 30 min, performed twice to ensure adequate dissociation. The resulting cell suspension was filtered through a 45-μm strainer and centrifuged at 500 × g for 3 min. Dissociated skin-derived cells were resuspended in freezing medium, transferred into cryovials, and stored at −80 °C for long-term preservation.

**Single-cell cDNA and library preparation**

Single-cell cDNA synthesis and library construction were performed following the 10× Genomics protocols at Seoul National University College of Medicine (Seoul, Republic of Korea). For samples PSO#1–4, single-cell suspensions were pooled prior to processing and loaded onto the Chromium Controller (10× Genomics) using the Chromium Next GEM Single Cell 5′ Reagent Kits v2 (CG000331, Rev D) according to the manufacturer’s protocol. For samples PSO#5–7, automated library preparation was conducted using the Chromium Connect system with the Chromium Next GEM Automated Single Cell 5′ Reagent Kits v2 (10× Genomics, USA), according to the manufacturer’s instructions (10× Genomics 5′ v2 Automation, CG000384, Rev C). In both protocols, single cells were encapsulated in Gel Beads-in-Emulsion (GEMs), where barcoded gel beads containing unique molecular identifiers (UMIs) and oligo-dT primers were copartitioned with individual cells. Within each GEM, mRNA transcripts were captured and reverse transcribed into cDNA, during which both a cell-specific barcode and a UMI were incorporated. The resulting cDNA was then PCR-amplified to generate sufficient material for downstream processing. For SNP-based demultiplexing of pooled samples, genomic DNA (gDNA) was extracted from paired PBMCs (average 1 × 10⁶ cells) using the QIAamp DNA Mini Kit (51306, QIAGEN), following the manufacturer’s protocol. The concentration and quality of extracted gDNA were assessed using a NanoDrop spectrophotometer (ND-ONEC-W, NanoDrop One/OneC, Thermo Fisher Scientific). Single-cell library quality and concentration were evaluated using the Agilent 4150 TapeStation system (Agilent Technologies). Final sequencing was performed on the Illumina NovaSeq 6000 platform.

**Preprocessing of scRNA-seq data**

Raw FASTQ files generated from Illumina sequencing were aligned to the human reference transcriptome (GRCh38) using Cell Ranger (v7.1.0, 10× Genomics). The resulting gene expression matrices were imported into Seurat (v4.9.9) within R (v4.2.1) for downstream scRNA-seq analysis^1^. Sample demultiplexing of pooled libraries was performed using Demuxalot with matched genomic DNA as reference, following the default parameters ^2^. To exclude doublets and multiplets, scDblFinder was applied with default settings^3^. Cells were retained if they expressed >200 genes and had <25% mitochondrial gene content. To account for technical variation across experiments, datasets were integrated using scVI^4^, with each library treated as a batch. Highly variable genes (HVGs) were identified using the Seurat’s default method after normalization, and the same batch key was applied during HVG selection and scVI modeling. Unsupervised clustering was performed using the Louvain algorithm, and cell embeddings were visualized via Uniform Manifold Approximation and Projection (UMAP). Marker genes for each cluster were identified using the FindAllMarkers function, and cell types were manually annotated based on known canonical markers. Data visualization and statistical analyses were performed in Python (v3.10) using matplotlib (v3.10.3) and scikit-learn (v1.6.1). All statistical tests were two-sided, and p-values < 0.05 were considered statistically significant.

**SCENIC analysis**

To infer cell type–specific gene regulatory networks, we applied the SCENIC (single-cell regulatory network inference and clustering) pipeline using the pySCENIC (v0.12.1) implementation^5^. Gene regulatory links were inferred using the GRNBoost2 algorithm, and motif enrichment was performed using the default cisTarget motif rankings. Regulon activity was quantified using AUCell based on the ranked expression of genes in each regulon. These AUCell-derived regulon activity scores served as a basis for ranking transcription factors and comparing their activity patterns between M1 and M2 macrophage subsets.

**Cell viability**

Cell viability was assessed using the Cell Counting Kit-8 (CCK-8; Dojindo) according to the manufacturer’s instructions. RAW 264.7 macrophages were seeded in 96-well plates at a density of 1 × 10^4^ cells per well and allowed to adhere overnight. Cells were then treated with the FABP4 inhibitor at concentrations of 0.1, 1, 10, and 100 μM for 24 hours.

Following treatment, 20 μL of CCK-8 solution was added to each well and incubated at 37°C for 2 hours. Absorbance was measured at 450 nm using a microplate reader (Bio-Rad). Cell viability was calculated as a percentage relative to untreated control cells. All experiments were performed in triplicate.

**References**

1.	Stuart T, Butler A, Hoffman P, et al. Comprehensive Integration of Single-Cell Data. *Cell*. Jun 13 2019;177(7):1888-1902 e21. doi:10.1016/j.cell.2019.05.031

2.	Rogozhnikov A, Ramkumar P, Shah K, Bedi R, Kato S, Escola GS. Demuxalot: scaled up genetic demultiplexing for single-cell sequencing. *bioRxiv*. 2021:2021.05.22.443646. doi:10.1101/2021.05.22.443646

3.	Germain PL, Lun A, Garcia Meixide C, Macnair W, Robinson MD. Doublet identification in single-cell sequencing data using scDblFinder. *F1000Res*. 2021;10:979. doi:10.12688/f1000research.73600.2

4.	Lopez R, Regier J, Cole MB, Jordan MI, Yosef N. Deep generative modeling for single-cell transcriptomics. *Nat Methods*. Dec 2018;15(12):1053-1058. doi:10.1038/s41592-018-0229-2

5.	Aibar S, Gonzalez-Blas CB, Moerman T, et al. SCENIC: single-cell regulatory network inference and clustering. *Nat Methods*. Nov 2017;14(11):1083-1086. doi:10.1038/nmeth.4463

**Supplementary Tables**

Supplementary Table 1. Mouse primer sequences used in the experiments.

| Gene | Primers (5′-3′) | |
| --- | --- | --- |
|  | Forward | Reverse |
| *gapdh* | GTCTCCTCTGACTTCAACAGCG | ACCACCCTGTTGCTGTAGCCAA |
| *fabp4* | TTTCTTTCAAACTGGGCGTG | CATTCCACCACCAGCTTGTC |
| *Il17a* | ATCCCTCAAAGCTCAGCGTGTC | GGGTCTTCATTGCGGTGGAGAG |
| *Il17f* | AACCAGGGCATTTCTGTCCCAC | GGCATTGATGCAGCCTGAGTGT |
| *Il1β* | TGTAATGAAAGACGGCACACC | TCTTCTTTGGGTATTGCTTGG |
| *tnfα* | TCTTCTCATTCCTGCTTGTGG | GGTCTGGGCCATAGAACTGA |
| *CD11c* | TGCCAGGATGACCTTAGTGTCG | CAGAGTGACTGTGGTTCCGTAG |
| *Mincle* | CCAAGTGCTCTCCTGGACGATA | CTGATGCCTCACTGTAGCAGGA |
| *iNOS* | ACTCAGCCAAGCCCTCACCTAC | TCCAATCTCTGCCTATCCGTCTCG |
| *IRF-5* | ATCTACGAGGTCTGCTCCAA | CAGGCTCTGTGATGCTCAG |
| *YM1* | TACTCACTTCCACAGGAGCAGG | CTCCAGTGTAGCCATCCTTAGG |
| *Arg1* | CAGAAGAATGGAAGAGTCAG | CAGATATGCAGGGAGTCACC |
| *CD206* | CCTATGAAAATTGGGCTTACGG | CTGACAAATCCAGTTGTTGAGG |
| *IRF4* | GAACGAGGAGAAGAGCGTCTTC | GTAGGAGGATCTGGCTTGTCGA |

Supplementary Table 2. Clinical characteristics of patients with psoriasis included in the scRNA-seq analysis.

| ***cohort*** | **Sample ID** | ***character*** | ***Age*** | ***Sex*** | ***subtype*** | **Disease duration (months)** | **Lesion location** | **PASI** | **previous treatments** |
| --- | --- | --- | --- | --- | --- | --- | --- | --- | --- |
| *Severance Hospital* | *Pso1* | *lesional* | *31* | *M* | *plaque* | *144* | back | 12.2 | unknown |
| *Severance Hospital* | *Pso2* | *lesional* | *25* | *M* | *plaque* | *8* | arm | 13 | unknown |
| *Severance Hospital* | *Pso3* | *lesional* | *20* | *F* | *plaque* | *108* | trunk | 7.2 | unknown |
| *Hanyang University* | *Pso4* | *lesional* | *33* | *F* | *plaque* | *324* | abdomen | 17.3 | unknown |
| *Hanyang University* | *Pso5* | *lesional* | *41* | *M* | *plaque* | *120* | leg | 14.1 | unknown |
| *Hanyang University* | *Pso6* | *lesional* | *22* | *F* | *plaque* | *120* | back | 12.3 | unknown |
| *Hanyang University* | *Pso7* | *lesional* | *38* | *M* | *plaque* | *300* | back | 25.8 | unknown |

**Supplementary Figures**

**Fig. S1 Single-cell transcriptomic landscape of psoriatic skin reveals macrophage polarization imbalance**

A. Uniform Manifold Approximation and Projection (UMAP) visualization of integrated single-cell RNA sequencing data from healthy control (HC) and psoriatic (PSO) skin samples.
Left panel: cells are colored according to annotated cell types, including myeloid cells, epithelial cells, fibroblasts, T cells, pericytes, vascular endothelial cells, lymphatic endothelial cells, mast cells, and melanocytes. Right panel: cells are colored by clinical condition (PSO, red; HC, blue), demonstrating disease-specific cellular distribution patterns.

B. Feature plots showing the expression of representative marker genes used for cell-type annotation across the UMAP embedding. Keratinocyte markers (KRT14, KRT1), fibroblast marker (COL1A1), pericyte marker (RGS5), T-cell marker (CD3D), mast cell marker (IL1RL1), melanocyte marker (MLANA), vascular endothelial marker (PECAM1), lymphatic endothelial marker (LYVE1), and myeloid lineage markers (AIF1, CD68, CD1C) are displayed. Color scale indicates normalized expression levels.

C. Volcano plot illustrating differentially expressed genes between M1 and M2 macrophage subsets. The x-axis represents log₂ fold change, and the y-axis indicates −log₁₀ adjusted p-value. Red dots denote genes enriched in M1 macrophages, while blue dots indicate genes enriched in M2 macrophages (min.pct > 0.1, adjusted p < 0.05). Selected representative genes are labeled.

**Fig. S2 FABP4 expression is increased in psoriasiform mouse skin and localizes to dermal macrophages in mouse and human lesional skin**

A. Comparative study between the normal and psoriasis skin showed upregulation of FABP4 in psoriatic skin. B. Quantitative analysis reveals the upregulation of FABP4 in psoriatic skin (n=3 biological replicates/groups). Data are presented as mean ± SEM; Unpaired t-test. NS: normal skin, PsO: psoriasis; scale bar: 100 μm.

C. Immunostaining analysis of FABP4 in the skin of imquimod-induced psoriasis mouse model. Designated part of magnified 2X and then white arrow showed FABP4 positive macrophage cell in mouse psoriasis skin (n=5 in each group). FABP4: Fatty acid binding protein 4, DAPI: 4′,6-diamidino-2-phenylindole, HC: Healthy Control, PSO: psoriasis

D. The representative immunofluorescence manifestations. Immunostaining analysis revealed distinct FABP4 localization in papillary dermis of psoriasis skin. Designated part of magnified 4X and then white arrow showed FABP4 positive macrophage cell in human psoriasis skin (n=3 in each group).

**Fig. S3 FABP4 inhibition restores PPARG transcriptional activity in psoriasis skin**

PPARG transcriptional activity in skin tissue lysates was measured using a PPARG-specific ELISA-based transcription factor activity assay kit according to the manufacturer’s instructions. Nuclear extracts were prepared from each experimental group as indicated. Individual dots represent independent biological replicates (n as indicated in the Methods), and bars denote mean ± SEM. Statistical significance was determined using one-way ANOVA followed by Tukey’s multiple comparisons test. *P < 0.05, **P < 0.01, ***P < 0.001.

**Fig. S4. Effects of FABP4 inhibitor on the viability of RAW 264.7 macrophages.**

RAW 264.7 cells were treated with various concentrations of the FABP4 inhibitor (0.1, 1, 10, and 100 μM) for 24 hours. Cell viability was assessed using the CCK-8 assay. No significant reduction in viability was observed at concentrations up to 10 μM, whereas a marked decrease was detected at 100 μM, indicating cytotoxicity at higher concentrations. Data are presented as mean ± SEM from three independent experiments. Statistical significance was determined using one-way ANOVA followed by Tukey’s multiple comparisons test. *P < 0.05, **P < 0.01, ***P < 0.001.

**Fig.S5 Representative flow cytometry dot plots for macrophage polarization analysis in mouse ear skin.**
Representative flow cytometry dot plots showing CD11c⁺ and CD206⁺ populations within viable CD45⁺F4/80⁺ macrophages isolated from mouse ear skin. CD11c⁺ and CD206⁺ populations were analyzed within the viable CD45⁺F4/80⁺ macrophage gate and interpreted as M1-associated/pro-inflammatory and M2-associated macrophage populations, respectively. Representative plots were exported using identical display settings across groups, and quantitative analysis was performed using all gated events.
